# β-Cyclodextrins Decrease Cholesterol Release and ABC-Associated Transporter Expression in Smooth Muscle Cells and Aortic Endothelial Cells

**DOI:** 10.3389/fphys.2016.00185

**Published:** 2016-05-25

**Authors:** Caroline Coisne, Dorothée Hallier-Vanuxeem, Marie-Christine Boucau, Johan Hachani, Sébastien Tilloy, Hervé Bricout, Eric Monflier, Daniel Wils, Michel Serpelloni, Xavier Parissaux, Laurence Fenart, Fabien Gosselet

**Affiliations:** ^1^EA 2465, Laboratoire de la Barrière Hémato-Encéphalique, Université d'ArtoisLens, France; ^2^Université Artois, CNRS, Centrale Lille, ENSCL, Université Lille, UMR 8181, Unité de Catalyse et de Chimie du Solide (UCCS)Lens, France; ^3^ROQUETTE, Nutrition DirectionLestrem, France

**Keywords:** aortic endothelial cells, atherosclerosis, ABCA1, ABCG1, cholesterol, methylated β-cyclodextrins, reverse cholesterol transfer, smooth muscle cells

## Abstract

Atherosclerosis is an inflammatory disease that leads to an aberrant accumulation of cholesterol in vessel walls forming atherosclerotic plaques. During this process, the mechanism regulating complex cellular cholesterol pools defined as the reverse cholesterol transport (RCT) is altered as well as expression and functionality of transporters involved in this process, namely ABCA1, ABCG1, and SR-BI. Macrophages, arterial endothelial and smooth muscle cells (SMCs) have been involved in the atherosclerotic plaque formation. As macrophages are widely described as the major cell type forming the foam cells by accumulating intracellular cholesterol, RCT alterations have been poorly studied at the arterial endothelial cell and SMC levels. Amongst the therapeutics tested to actively counteract cellular cholesterol accumulation, the methylated β-cyclodextrin, KLEPTOSE® CRYSMEβ, has recently shown promising effects on decreasing the atherosclerotic plaque size in atherosclerotic mouse models. Therefore we investigated *in vitro* the RCT process occurring in SMCs and in arterial endothelial cells (ABAE) as well as the ability of some modified β-CDs with different methylation degree to modify RCT in these cells. To this aim, cells were incubated in the presence of different methylated β-CDs, including KLEPTOSE® CRYSMEβ. Both cell types were shown to express basal levels of ABCA1 and SR-BI whereas ABCG1 was solely found in ABAE. Upon CD treatments, the percentage of membrane-extracted cholesterol correlated to the methylation degree of the CDs independently of the lipid composition of the cell membranes. Decreasing the cellular cholesterol content with CDs led to reduce the expression levels of ABCA1 and ABCG1. In addition, the cholesterol efflux to ApoA-I and HDL particles was significantly decreased suggesting that cells forming the blood vessel wall are able to counteract the CD-induced loss of cholesterol. Taken together, our observations suggest that methylated β-CDs can significantly reduce the cellular cholesterol content of cells forming atherosclerotic lesions and can subsequently modulate the expression of ABC transporters involved in RCT. The use of methylated β-CDs would represent a valuable and efficient tool to interfere with atherosclerosis pathogenesis in patients, nonetheless their mode of action still needs further investigations to be fully understood and finely controlled at the cellular level.

## Introduction

Vascular dysfunction represents a key event in the pathogenesis of atherosclerosis, a chronic disease characterized by the increased deposition of cholesterol in the artery intima. In the first steps of the disease, inflammatory processes lead to the recruitment of cholesterol-laden macrophages in the artery wall that form the so-called foam cells filled with numerous cholesterol ester droplets (Allahverdian and Francis, 2010). An additional mechanism for the generation of these foam cells in atherosclerotic plaques is the conversion of smooth muscle cells (SMCs) from the artery wall into foam cells (Allahverdian et al., 2014). Studies focusing on the balance between lipid accumulation and lipid removal from cells have highlighted that the complex cholesterol metabolism is controlled by the reverse cholesterol transport (RCT) which is mainly dependent on the expression and activity of three prominent proteins promoting the cellular cholesterol efflux (Phillips, 2014). Two of them belong to the ATP-binding cassette (ABC) transporter family comprising 48 members in humans (Kim et al., 2008) and correspond to the first member of the sub-family A (ABCA1) and the first member of the sub-family G (ABCG1). Studies in macrophages revealed that apoA-I initially interacts with ABCA1 to form a partially lipidated discoidal complex that subsequently interacts with ABCG1 in order to get additional cholesterol and to generate spherical lipoprotein particles, the high density lipoproteins (HDL) (Gelissen et al., 2006). The third protein involved in the RCT is the scavenger receptor class B member 1 (SR-BI, encoded by *SCARB1* gene) which mediates bidirectional cholesterol exchanges between cell membrane and HDL. We and others have investigated the expression pattern and functionality of SR-BI and these two ABC transporters at the macrophage level as well as their abilities to initiate and generate HDL (Linsel-Nitschke and Tall, 2005; Wang et al., 2007; Mahmood et al., 2013; Phillips, 2014). However the RCT has received little attention at the arterial endothelial cell and SMC levels (Allahverdian and Francis, 2010).

These studies clearly highlight the importance to characterize the role of ABCA1, ABCG1, and SR-BI in order to prevent or to develop targeted therapies to treat cardiovascular and metabolic diseases. For this reason, current therapeutic perspectives in atherosclerosis aim at promoting cholesterol efflux by a process resulting in an increase in ABCA1 and ABCG1 expression that generates higher amount of HDL. For example, activation of the Liver X receptor (LXR) signaling pathway regulating the ABCA1/ABCG1 expression have been shown to promote macrophage RCT (Naik et al., 2006) and decrease atherosclerosis in mouse models (Terasaka et al., 2003). Another efficient approach consists in using molecules able to deplete the cellular cholesterol content, for instance, the β-cyclodextrin subset (β-CD). This latter one is member of the cyclodextrin (CD) family which is composed of cyclic oligosaccharides prepared from starch after an enzymatic cleavage. Amongst CDs, β-CD consists of 7 D-glucopyranose units which possess 21 hydroxyl moieties. Its shape is a conical cylinder whose inner surface is hydrophobic and outer surface hydrophilic (Mahammad and Parmryd, 2015). These hydroxyl groups can be modified via specific routes conferring particular biochemical and biological properties to the CDs. For these reasons, β-CD family is widely used in the pharmaceutical field to improve dissolution rate, chemical stability and drug bioavailability. When β-CD is partially methylated, the resulting compounds are named methylated β-CDs and are able to interact with cell membranes and thus influence their cholesterol/phospholipid content (Mahammad and Parmryd, 2015). This process remains however partially understood but could still be promising for the treatment of patients suffering from abnormal cholesterol storage diseases such as in the Niemann-Pick C (NPC) disease. This disorder is characterized by an abnormal lysosomal lipid storage caused by a genetic mutation in genes coding for proteins involved in the intracellular cholesterol trafficking. *In vitro* and *in vivo* studies have demonstrated that CDs are able to trap membrane-stored cholesterol rendering them promising therapeutic agents for novel treatments in patients with NPC disease (Vance and Karten, 2014). Additionally, cyclodextrins have recently shown therapeutic potential in Alzheimer disease (AD) which is characterized by a cerebral accumulation of amyloid peptides (Gosselet et al., 2013) and by a disturbed brain cholesterol homeostasis (Gosselet et al., 2014). In a mouse model of AD, Yao et al. demonstrated that hydroxypropyl-β-CD reduces amyloid production and clearance probably via an ABCA1-mediated process (Yao et al., 2012).

All these data have recently provoked an increased interest for the use of cholesterol-sequestrating agents in the atherosclerosis context. The anti-atherosclerotic action of KLEPTOSE® CRYSMEB, a family member of the methylated-β-CDs, has been highlighted in atherosclerotic mouse models demonstrating that this particular CD modifies HDL-cholesterol levels and decreases cholesterol accumulation in atherosclerotic lesions (Montecucco et al., 2015). However, no study focusing on the effect of this CD on the RCT process in arterial endothelial cells and in SMCs has been performed so far. Here we investigate *in vitro* the effects of several methylated β-CDs including KLEPTOSE® CRYSMEβ on cholesterol regulation in aortic bovine arch endothelial (ABAE) cells and SMCs. CDs were able to extract membrane cholesterol from both cell types resulting in a decreased cellular cholesterol content. This decrease in cellular cholesterol diminished the expression of ABCA1 and ABCG1, but not SR-BI expression, and reduced cellular cholesterol release. In both cell types, treatment with the LXR agonist T0901317 increased ABC transporter expressions and promoted cholesterol release to ApoA-I and HDL. However when cells were depleted in cholesterol by methylated β-CD treatment, T0901317 only promoted cholesterol efflux in ABAE cells. Therefore, by reducing the cellular cholesterol content, our results demonstrate that methylated β-CDs can efficiently interfere with the pathological process of atherosclerosis. Novel *in vivo* and *in vitro* approaches would be necessary to further characterize the therapeutic potential of these molecules to treat atherosclerosis.

## Materials and methods

### Cyclodextrins (CDs)

Except for Rameβ which was provided by Sigma-Aldrich (Lyon, France), the various cyclodextrins (CDs) were synthesized, purified, modified and lyophilized by Roquette Frères (Lestrem, France). Before the experiments, they were stored in a dry place at room temperature. For experiments they were freshly solubilized in Dulbecco's modified Eagle's medium (DMEM). Table 1 recapitulates the different CDs used in this study and their methylation degree (i.e., the number of methyl groups per glucopyranose unit) and their molecular weight. The methylation degrees were determined by ^1^H NMR spectroscopy and MALDI-TOF experiments.

**Table 1:** **Description of the different CDs used in this study**.

**Name**	**Methylation degree**	**Molecular weight (g/mol)**
β-CD	0	1135
Methyl-β-CD	0.23	1157
KLEPTOSE® CRYSMEβ	0.57	1191
Rameβ	1.8	1311

### Chemicals

ApoA-I and HDL were purchased from PROSCI Incorporated (Poway, CA, USA). Bovine serum albumin (BSA) and T0901317 were purchased from Sigma-Aldrich. T0901317 was dissolved and used as previously described (Saint-Pol et al., 2012). [^3^H]-cholesterol (43 Ci/mmol) was purchased from PerkinElmer Life and Analytical Sciences (Waltham, MA, USA).

### Cell culture

This study was carried out in accordance with the recommendations of the French veterinary council's guide (approval n° B62-498-5). Aortic bovine arch endothelial cells (ABAE) and bovine SMCs were extracted, purified and characterized as previously published (Dehouck et al., 1994, 1997; Vandenhaute et al., 2011). Cells were cultured in 12 and 24-well plates coated with porcine skin gelatin (Sigma-Aldrich). ABAE and SMCs were seeded at 25,000 and 50,000 cells per well, respectively and cultured in DMEM supplemented with 2 mM glutamine, 50 μg/mL gentamycin, 1 ng/mL basic fibroblast growth factor (bFGF) and with 10% (v/v) horse serum (HS) and 10% calf serum (CS) for ABAE and with 20% fetal calf serum (FCS) for SMCs. When cells reached 60-80% of confluence, treatment with CDs was initiated.

### Cell death

Cells were incubated for 24 h with increasing CD concentrations (from 0.1 to 25 mM) in DMEM supplemented with bFGF. The control condition consisted in DMEM with bFGF. Cell death was then quantified using the Cyto-ToxONE Homogeneous Membrane Integrity Assay (Promega, Madison, WI, USA) following supplier's instructions. Briefly, this kit measures the release of the lactate dehydrogenase (LDH) by cells with damaged membranes. Colorimetric values were measured using the Synergy™ H1 (Biotek, Shoreline, WA, USA) and data represent the percentage of cell death compared with a 100% cell death control which corresponds to the total lysis of a cell sample using the lysis buffer provided in the kit.

### mRNA extraction and real time RT-PCR analysis

Twenty-four hours after treatment with 1 mM CDs supplemented or not with 10 μM T0901317, ABAE and SMCs were rinsed twice with cold phosphate-buffered saline (8 g/L NaCl, 0.2 g/L KCl, 0.2 g/L KH_2_PO_4_, 2.87 g/L NA_2_HPO_4_ (12H_2_O), pH 7.4) and then lysed in 200 μL RLT lysis buffer (Qiagen, Venlo, the Netherlands). Three wells per condition were pooled and mRNA purification was performed using the RNeasy total RNA extraction kit (Qiagen) following manufacturer's instructions. The purity and the concentration of the extracted mRNA were assessed by measuring the absorbance at 260, 280, and 320 nm using the Tek3 microplate reader protocol (Synergy™ H1, Biotek). Only mRNA samples with high purity values (>2) were used in our studies. For each condition, cDNAs were obtained from 0.250 mg of mRNA using iScript™ Reverse Transcription Supermix (BioRad, Hercules, CA, USA) according to manufacturer's instructions. Real-time PCR experiments were performed using the Sso Fast EvaGreen Master Mix kit (BioRad) and custom primers (listed in Table 2) which were used in our previous studies (Saint-Pol et al., 2012, 2013). For each primer, amplification was carried out for 40 cycles with an annealing temperature of 60°C using a CFX96 thermocycler (BioRad). The amplification efficiency was determined for each primer pair and used in the calculation method (CFX Manager, BioRad). Melting curve analysis was performed after amplification cycles in order to check the specificity/purity of each amplification. Gene expression levels were evaluated according to the ΔΔCt method and were normalized against ß*-ACTIN*.

**Table 2:** **Primers designed for real time RT-PCR**.

**mRNA**	**Species**	**F/R**	**Sequences**	**Accession Number**
ABCA1	Bos taurus	F	5′-gtgtctcgcctgttctcag-3′	NM_001024693
		R	5′-gaaacatcacctcctgccg-3′	
ABCG1	Homo sapiens	F	5′-gaggaagaaaggatacaagacc-3′	BC029158
		R	5′-gtcagtatctccttgaccattt-3′	
SCARB1	Bos taurus	F	5′-cccttaatccacctcatcaatc-3′	NM_174597
		R	5′-gaagtttttgacccctgtgaac-3′	
ß-ACTIN	Bos taurus	F	5′-gcacaggcctctcgccttcg-3′	NM_173979
		R	5′-acatgccggagccgttgtcg-3′	

*From left to right: mRNA targeted for amplification, species, sequences (Forward (F), Reverse (R) primer), and mRNA accession numbers in NCBI database*.

### Protein extraction and analysis

After a 24 h treatment with CDs (1 mM) supplemented or not with 10 μM T0910317, cells were washed twice with cold PBS and lysed in 100 μL RIPA buffer (50 mM Tris-HCl (pH 7.4), 150 mM NaCl, 0.25% deoxycholic acid, 1% NP-40, and 1 mM EDTA) supplemented with protease/phosphatase inhibitors (Sigma-aldrich). Six wells of a 12-well plate per condition were pooled. Protein concentration was then assessed using Bradford method (Biorad). Afterwards, proteins (40 μg/condition for ABAE and 20 μg/condition for SMCs) were separated on a 4–15% sodium dodecyl sulfate polyacrylamide gel (BioRad) and electrotransferred onto nitrocellulose membranes (GE Healthcare, Barrington, IL, USA). After a 90 min incubation in blocking buffer (25 mM Tris-HCl (pH 8.0), 125 mM NaCl, 0.1% Tween 20 and 5% skimmed milk) at 37°C, membranes were incubated at 4°C overnight with either mouse anti-ABCA1 (2 μg/mL, ABCAM, Paris, France) or rabbit anti-ABCG1 (5 μg/mL, ABCAM) or rabbit anti-SR-BI (0.4 μg/mL, ABCAM) and mouse anti-β-ACTIN antibody (1 μg/mL, Sigma-aldrich). Afterwards, membranes were washed three times in blocking buffer and incubated with their appropriate horseradish peroxidase (HRP)-conjugated secondary antibody (Dako, Glostrup, Denmark) for 1 h at room temperature. HRP was detected with an enhanced chemiluminescence kit (GE Healthcare) and revealed on chemiluminescence-sensitive films (GE Healthcare). Optical band densities were measured using TotalLab TL 100 1D Gel Analysis software (Non-linear Dynamics, Newcastle, UK). In these experiments β-ACTIN was used as the loading control.

### Cholesterol depletion and cellular cholesterol efflux from ABAE and SMCs

First, [^3^H]-cholesterol (0.5 μCi/mL) was incorporated in the serum (10% HS/CS for ABAE and 20% FCS for SMCs, as described above) for 6 h at 37°C. The resulting radiolabeled serum was then supplemented with 1 ng/mL bFGF, 2 mM glutamine, and 50 μg/mL gentamycin in order to obtain a [^3^H]-cholesterol enriched-medium in which cells in 12-well plates were cultured for 36 h. Afterwards, cells were rinsed twice with DMEM/0.1% Bovine serum albumin (BSA) and cultured in 1.4 mL DMEM/1 ng/mL bFGF supplemented or not with 1 mM CDs for 24 h at 37°C in 5% CO_2_. After a 2 h incubation period, 500 μL of medium were removed and the amounts of [^3^H]-cholesterol measured in order to evaluate the effect of CDs on the cell membrane extraction of [^3^H]-cholesterol. To this aim, medium samples were centrifuged (4 min, 4000 rpm, 4°C) to remove cellular debris and the amounts of [^3^H] measured with a scintillation counter (TriCarb 2100TR, PerkinElmer).

Cell extracted [^3^H]-cholesterol by CDs and cellular cholesterol amounts, expressed in disintegration per minute (DPM), were obtained using the following equation:

$$\begin{array}{l} \text{Cell extracted}{[}^{3}\text{H}]-\text{cholesterol by CDs}\\ \;={\text{DPM}}_{\text{media }2\text{ h}}*(1.4/0.5) \end{array}$$

Values were then converted in percentage and compared with the control condition (no CD supplementation in medium) which was set to 100%. To evaluate the effect of the CDs on the RCT, cells were treated 24 h with different CDs at 1 mM in [^3^H]-cholesterol enriched-medium. Afterwards cells were rinsed twice with DMEM/0.1% BSA and cellular cholesterol efflux was assessed for 6 h in absence or presence of cholesterol acceptors (20 μg/mL ApoA-I or 50 μg/mL HDL) in DMEM/0.1% BSA. At the end of this step, cell medium supernatants were collected and centrifuged (4 min, 4000 rpm, 4°C) whereas cells were lysed in 1% Triton X-100 in PBS after 4 washes in cold PBS. Ultimately ^3^H-radioactivity was measured in supernatants and cell lysates using the scintillation counter described above. Protein concentration was also assessed in each sample using the Bradford method and final results in **Figures 6**, **9** were expressed as the amount of DPM measured/sample divided by its protein concentration in microgram.

In **Figure 7**, results were presented as previously described (Saint-Pol et al., 2012, 2013; Kuntz et al., 2015) and consist in calculating the percentage of cholesterol efflux while taking into account the remaining cellular cholesterol load, following this formula:

$$\begin{array}{l} \%\text{ cholesterol efflux}=({\text{DPM}}_{\text{MEDIUM}}/({\text{DPM}}_{\text{MEDIUM}}\\ \;+{\text{DPM}}_{\text{CELL LYSATE}}))\times 100 \end{array}$$

To assess the effect of T0901317 in ABAE cells and SMCs (**Figure 9**), this LXR-agonist was added in the media during Rameβ-treatment at a concentration of 10 μM. Then, cholesterol efflux was measured as described above. Control condition (medium alone, not supplemented with neither Rameβ nor T0901317 but only with adequate volume of DMSO) was set to 100%. Each experiment was performed in triplicate and repeated twice.

### Statistics

Statistical analyses were performed using the GraphPad Prism 5.01^®;^ software (Graphpad software, La Jolla, USA). Results were analyzed and compared together using appropriate post-hoc tests (see figure legend) where, ^*^p < 0.05; ^**^p < 0.01; ^***^p < 0.001; and ^*##*^*p* < 0.01.

## Results

### *ABCA1, ABCG1*, and *SCARB1* gene expression in abae and SMCs

**Figure 1:**
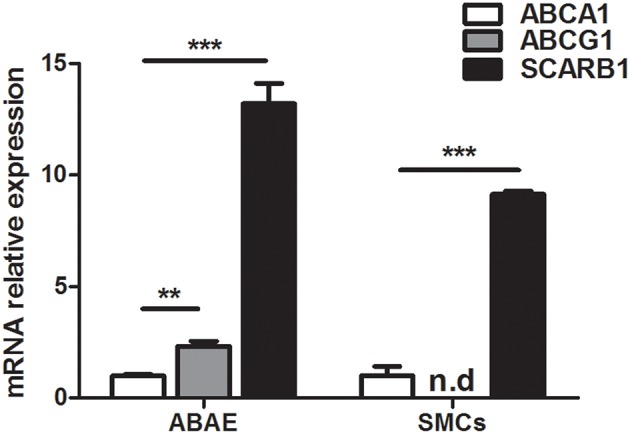
**mRNA expression of *ABCA1*, *ABCG1*, and *SCARB1* in ABAE and SMCs by real time RT-PCR**. Each bar represents the absolute quantification of the three studied gene. Used primers are listed in Table 1. ABCA1 expression level is used as the reference level and set to 1. ABCG1 expression was not detected in SMCs. Results correspond to the mean ± SD of three different experiments pooled from three wells. n.d: not detected. One-way ANOVA test followed by Bonferroni correction was performed in which ^***^p < 0.001; ^**^p < 0.01 when compared with the ABCA1 expression.

*ABCA1, ABCG1*, and *SCARB1* expressions were investigated in aortic endothelial cells (ABAE) and in SMCs with real time RT- PCR method using primers described in Table 2. Figure 1 shows that *ABCA1* and *SCARB1* expression was detected in both cell types whereas *ABCG1* was only found in ABAE not in SMCs. In our culture conditions (DMEM), we observed that the amount of *ABCG1* and *SCARB1* mRNA expressed in ABAE were 2.33-fold and 13.20-fold higher than the amount of *ABCA1* mRNA, respectively. In SMCs, *SCARB1* was expressed 9.14-fold more than *ABCA1*.

### Cellular cholesterol efflux from ABAE and SMCs

**Figure 2:**
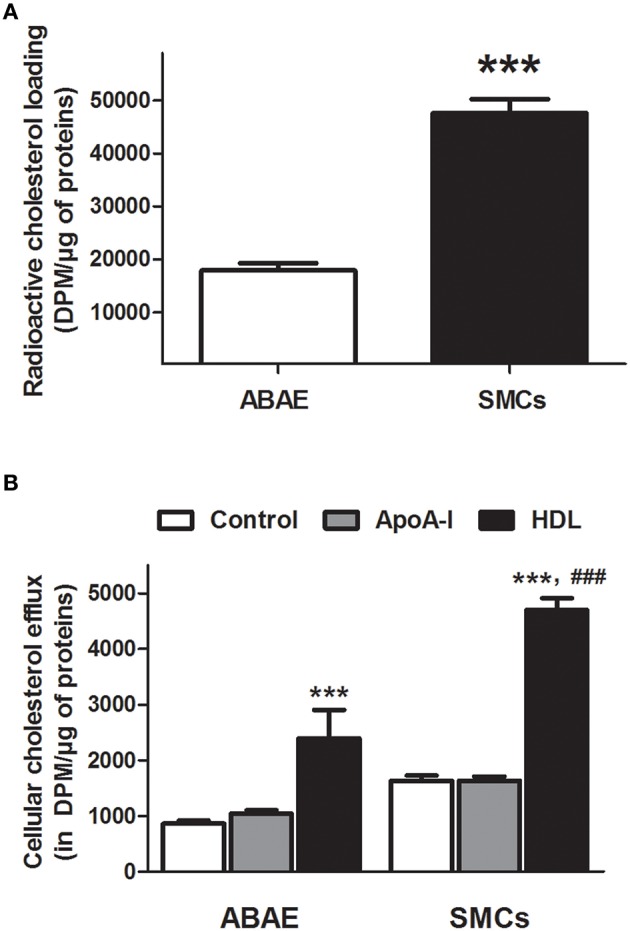
**Cholesterol efflux from ABAE and SMCs**. Cells were first labeled with [^3^H]-cholesterol (0.5 μCi/mL) for 36 h at 37°C and then equilibrated for 24 h in DMEM/0.05% BSA at 37°C. Cellular [^3^H]-cholesterol loads in DPM per μg of proteins were compared between ABAE and SMCs **(A)**. Data represent the mean ± SEM (in DPM/μg of proteins), with *n* = 6 from two sets of experiments. Paired-*t*-test was performed, ^***^p < 0.001 when compared with ABAE condition. [^3^H]-cholesterol released from both cell types was then measured 6 h after medium supplementation with either BSA (control condition) or cholesterol acceptors [ApoA-I (20 μg/mL) or HDL (50 μg/mL)] at 37°C **(B)**. Data represent the mean ± SEM (in DPM/μg of proteins), with *n* = 6 from two sets of experiments. One-way ANOVA test followed by Bonferroni correction was performed in which ^***^p < 0.001 refers to the control condition and ^*###*^*p* < 0.001 refers to the matched HDL condition in ABAE.

Figure 2A shows cholesterol accumulation in ABAE and SMCs. Cholesterol accumulated three-fold less in ABAE than in SMCs (17,918 ± 1319 DPM/μg of proteins vs. 47,672 ± 2633 DPM/μg of proteins, respectively). Cholesterol efflux was then assessed in ABAE and in SMCs in the absence (BSA only, control condition) or in the presence of cholesterol acceptors (BSA supplemented either with ApoA-I or with HDL) (Figure 2B). Without any cholesterol acceptor (control condition), the amount of cholesterol released from SMCs was more important than the one released from ABAE by two-fold (1635 vs. 865 DPM/μg of proteins, respectively). In the presence of either ApoA-I or HDL, the efflux was 1043 and 2395 DPM/μg of proteins, respectively in ABAE, and 1639 and 4701 DPM/μg of proteins, respectively in SMCs. These results suggest that in SMCs and in ABAE, ApoA-I does not promote cholesterol efflux from cell membrane in contrast to HDL as previously reported for other cell types (Saint-Pol et al., 2012, 2013). Additionally, when comparing the cholesterol efflux to HDL, our results shows that cholesterol efflux to HDL particles was much more effective from SMCs than from ABAE.

### Effects of the different methylated CDs on the cellular cholesterol content in ABAE and SMCs

Before assessing the effects of differentially methylated β-cyclodextrins on the cellular cholesterol content (RCT), we performed viability tests using increasing concentration of CDs (0.1, 0.5, 1, 2.5, 5, 10, and 25 mM). In comparison to SMCs, ABAE appeared more resistant to the same CD treatment as cell toxicity could only be detected at 5 mM for β-CD and Rameβ, and at 10 mM for the KLEPTOSE® CRYSMEβ and Methyl-β-CD (Table 3). In SMCs, no toxicity was detected below a concentration of 2.5 mM for any CD tested. Therefore to avoid any artifact due to CD-induced cell death (Kiss et al., 2010; Mahammad and Parmryd, 2015), the concentration of 1 mM was selected for further experiments.

**Table 3:** **Determination of the toxicity of the CDs**.

**Name**	**Toxic concentration threshold determined for ABAE (in mM)**	**Toxic concentration threshold determined for SMCs (in mM)**
β-CD	5	2.5
Methyl-β-CD	10	2.5
KLEPTOSE® CRYSMEβ	10	2.5
Rameβ	5	2.5

*ABAE and SMCs were treated for 24 h with increasing concentrations (0.1, 0.5, 1, 2.5, 5, 10, and 25 mM) of the appropriate CD (described in Table 1). Afterwards, the lactate dehydrogenase activity in cells was measured for each CD concentration and compared with controls in order to estimate cell death induced by CDs. Each experiment was performed in triplicate and repeated three times*.

**Figure 3:**
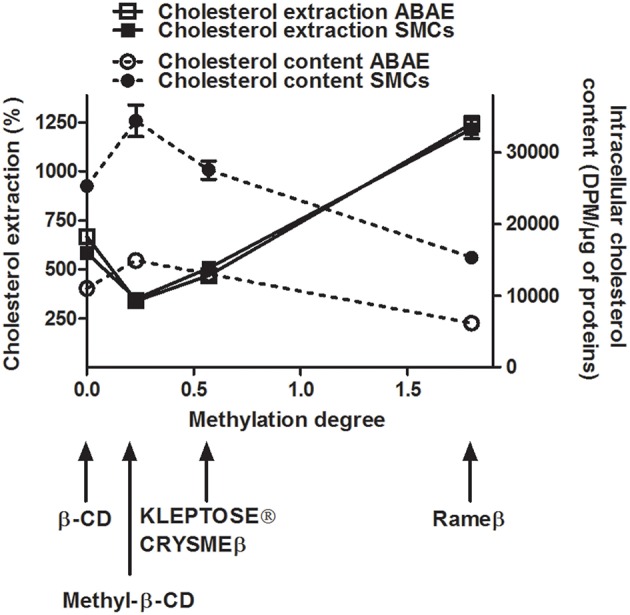
**Cellular cholesterol extraction (continuous lines) and cellular cholesterol content (dashed lines) in cyclodextrin-treated ABAE (open symbols) and SMCs (solid symbols)**. Cells were first labeled with [^3^H]-cholesterol (0.5 μCi/mL) for 36 h at 37°C and then equilibrated in DMEM/0.05% BSA for 24 h at 37°C. After a 2 h incubation period with different CDs (1 mM), the amounts of [^3^H]-cholesterol contained in the medium (in percentage) and within the cells (in DPM/μg of proteins) were measured. For cholesterol extraction data, all results referred to control condition (no CD, only DMEM) which is set to 100%. Data are expressed as the mean ± SEM (*n* = 15).

After 2 h of treatment with 1 mM of the appropriate CDs, we observed that, independently to the cell type, the β-CD (methylation degree 0, Table 1) was able to extract more than 580% of cholesterol when compared with the respective ABAE and SMC control condition (no CD treatment) (Figure 3). Methyl-β-CD (methylation degree 0.23), KLEPTOSE® CRYSMEβ (methylation degree 0.57) and Rameβ (methylation degree 1.8) were able to extract 350.4, 507.1, and 1216.9% of cholesterol, respectively. Despite the fact that ABAE accumulated less cholesterol than SMCs (Figure 2A), percentages of extracted-cholesterol were identical between both cell types for each evaluated CD. Therefore, we can conclude that cholesterol extraction rather depends on the methylation degree of the CDs and not on the cell type. As also shown in Figure 3, cholesterol cellular content in SMCs and in ABAE varied proportionally to the capture of cholesterol by CDs when compared with the untreated condition. In ABAE, β-CD and Rameβ decreased the cholesterol cellular content to 11,015 and 6162 DPM/μg of proteins (representing a decrease by 38.52 and 65.6%, respectively) whereas Methyl-β-CD and KLEPTOSE® CRYSMEβ decreased it to 14,892 and 13,022 DPM/μg of proteins, respectively (a decrease by 16.9 and 27.3%, respectively). In SMCs, β-CD and Rameβ decreased the cholesterol cellular content to 25,276 and 15,298 DPM/μg of proteins, respectively (by 47.0 and 67.9%, respectively) whereas Methyl-β-CD and KLEPTOSE® CRYSMEβ decreased it to 34,408 and 27,539 DPM/μg of proteins, respectively (by 42.2 and 27.3%, respectively).

### Effects of the methylated β-CDs on the reverse cholesterol transport

**Figure 4:**
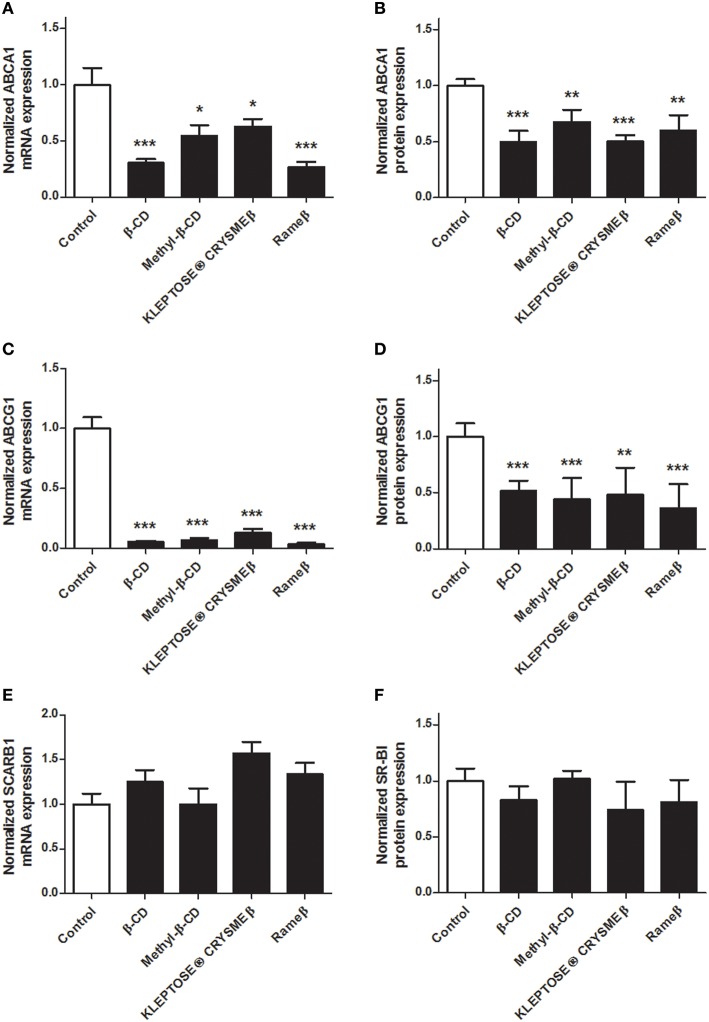
**Evaluation of the expressions of ABCA1, ABCG1, and SR-BI upon CD-treatment in ABAE**. Twenty-four hours after treatment (CDs 1 mM, solid bars), mRNA expressions of *ABCA1* **(A)**, *ABCG1* **(C)** as well as of *SCARB1* **(E)** were assessed and compared with control (DMEM treatment, open bar). Each bar represents the mRNA expression normalized against the housekeeping gene β*-ACTIN* ± SEM (*n* = 6). Control condition (without CD treatment) is used as the reference level and set to 1. A one-way ANOVA test followed by Dunnett's test for multiple comparisons was applied with ^*^p < 0.05; ^**^p < 0.01; ^***^p < 0.001 compared with control. Normalized protein expression levels for ABCA1 **(B)**, ABCG1 **(D)**, and SR-BI **(F)** were assessed by immunoblotting. β-ACTIN was used as the loading control. Results correspond to the optical density measured in three experiments. Control condition (without any CD treatment) is used as the reference level and set to 1.

**Figure 5:**
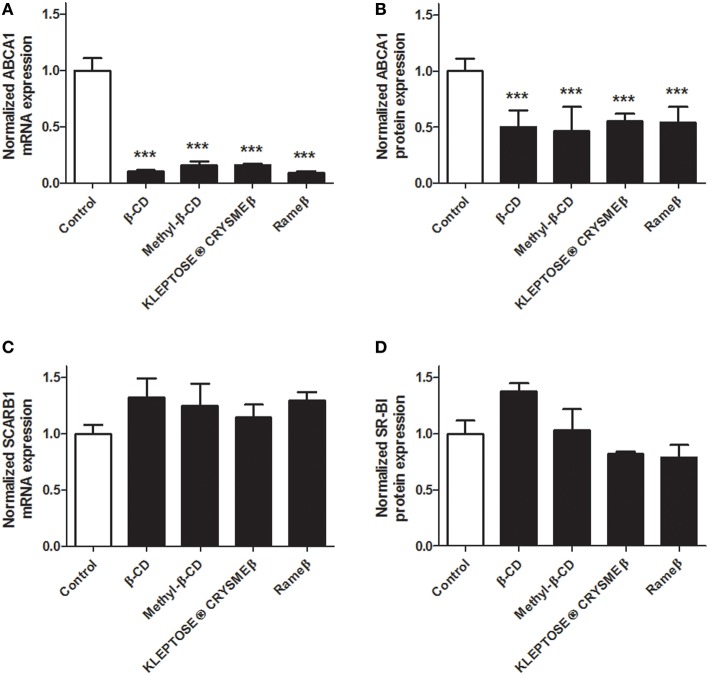
**Evaluation of the expressions of ABCA1 and SR-BI upon CD-treatment in SMCs**. Twenty-four hours after treatment (CDs 1 mM, solid bars), mRNA expressions of *ABCA1* **(A)** and *SCARB1* **(C)** were assessed and compared with control (DMEM treatment, open bar). Each bar represents the mRNA expression normalized against the housekeeping gene β*-ACTIN* ± SEM (*n* = 6). Control condition (without CD treatment) is used as the reference level and set to 1. A one-way ANOVA test followed by Dunnett's test for multiple comparisons was applied with ^***^p < 0.001 compared with control. Normalized protein expression levels for ABCA1 **(B)** and SR-BI **(D)** were assessed by immunoblotting. β-ACTIN was used as the loading control. Results correspond to the optical density measured in three experiments. Control condition (without any CD treatment) is used as the reference level and set to 1.

To determine whether the cholesterol depletion induced by CD treatment may impact the cellular cholesterol metabolism, transcriptional and protein expressions of key players of the reverse cholesterol transfer (RCT), i.e., ABCA1, ABCG1, and SR-BI, were investigated by real time RT-PCR and immunoblotting. In ABAE, mRNA expression of *ABCA1* and *ABCG1* was significantly down-regulated in all evaluated CD conditions (Figures 4A,C, respectively) compared with control. Such decrease in gene transcription was confirmed at the protein level for both ABCA1 (Figure 4B) and ABCG1 (Figure 4D). The expression of SR-BI was not modified by any CD treatment (Figures 4E,F). In SMCs, no expression of ABCG1 was detected at mRNA and protein levels, neither before nor after any CD treatment (data not shown). However, the transcriptional and protein expression of ABCA1 was significantly decreased for each tested CD condition (Figures 5A,B) compared with control. As previously observed in ABAE cells, the expression of SR-BI in SMCs was not significantly modified by any CD treatment (Figures 5C,D).

**Figure 6:**
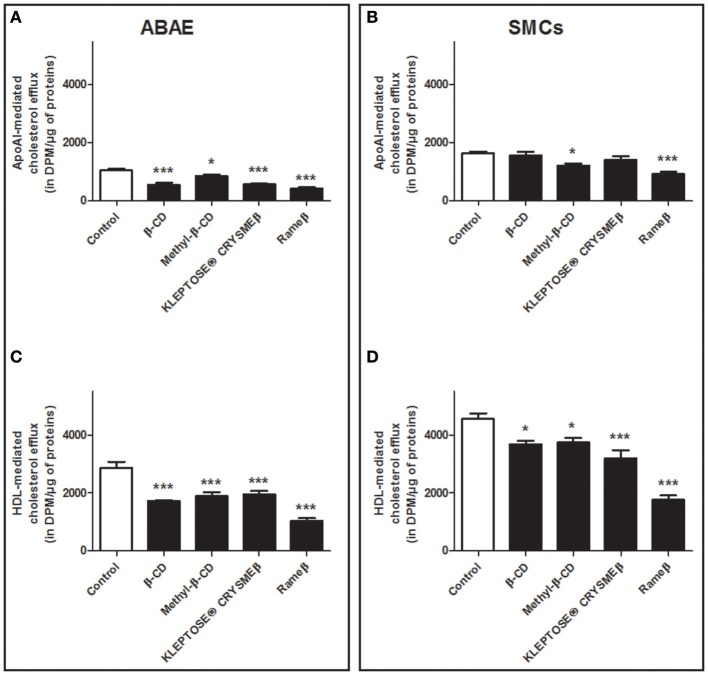
**Cholesterol release from ABAE (A,C) and from SMCs (B,D) upon CD-treatment**. Cells were first labeled with [^3^H]-cholesterol (0.5 μCi/mL) for 36 h at 37°C and then equilibrated in DMEM/0.05% BSA for 24 h at 37°C. [^3^H]-cholesterol released in the medium from both cell types was then measured 6 h after medium supplementation with cholesterol acceptors [ApoA-I (20 μg/mL) or HDL (50 μg/mL)] at 37°C. The radioactivity contained in the culture medium, that corresponds to the cholesterol released from cells was measured. Data are expressed as the mean ± SEM (in DPM/μg of proteins, *n* = 6 from two sets of experiments). Statistical analysis: a one-way ANOVA followed by Dunnett's test for multiple comparisons in which ^*^p < 0.05; ^***^p < 0.001 compared with the control condition (open bars) without any CD treatment.

Next, aiming at investigating the consequences of CD-mediated ABCA1/ABCG1 down-regulation on the cellular cholesterol efflux, ABAE and SMCs were labeled with [^3^H]- cholesterol and the cholesterol release, mediated via lipid-free apoA-I or HDL particles, was then measured (Figure 6). In ABAE, a significant decrease in the cholesterol efflux to ApoA-I (Figure 6A) and to HDL (Figure 6C) particles was observed. In fact, β-CD, Methyl-β-CD, KLEPTOSE® CRYSMEβ and Rameβ decreased the ApoA-I-mediated cholesterol release by 46.26, 18.41, 45.19, and by 59.07%, respectively (Figure 6A) and the HDL-mediated cholesterol efflux was reduced by 39.94, 34.25, 32.05, and by 63.75%, respectively (Figure 6C). In SMCs, except for methyl-β-CD and Rameβ conditions in which the decrease in cholesterol efflux reached 25.25 and 43.49% respectively, the other CDs did not significantly alter cholesterol release to ApoA-I particles (Figure 6B). On the contrary, cholesterol efflux to HDL was significantly decreased by β-CD, Methyl-β-CD, KLEPTOSE® CRYSMEβ and Rameβ, by 14.75, 15.12, 29.46, and by 60.21%, respectively (Figure 6D).

**Figure 7:**
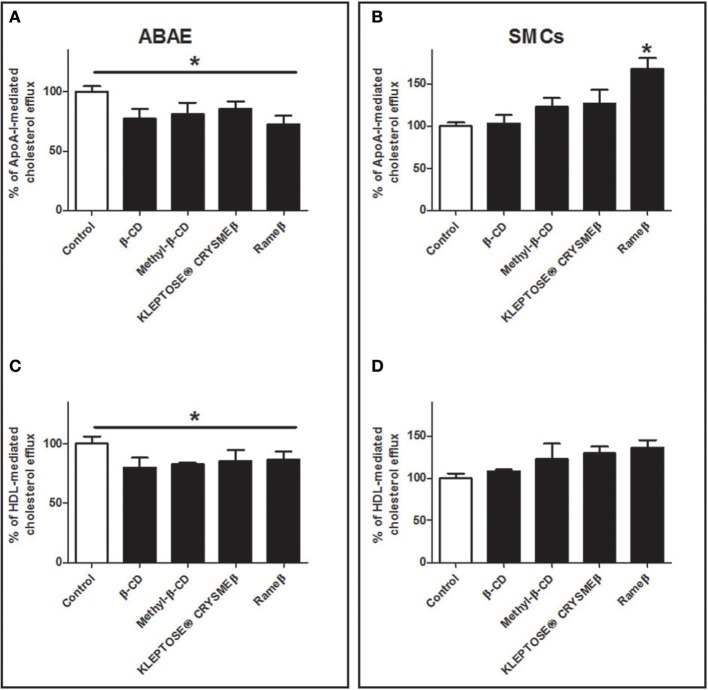
**Percentage of cholesterol release from ABAE (A,C) and from SMCs (B,D) upon CD-treatment taking into account the cholesterol load at the start of the experiments**. Results showed here are the same as the ones from Figure 6 but the cellular cholesterol content remained in cells after cholesterol efflux was taken into account in the calculation method. A one-way ANOVA test followed by Dunnett's test for multiple comparisons was applied with ^*^p < 0.05 compared with control condition.

It is now well-described that sterol concentration in cell membrane as well as cholesterol pool in whole body remain quite constant (Dietschy and Turley, 2004), therefore data from Figure 6 were additionally analyzed with another method consisting in taking into account the total cholesterol load in cell after CD-depletion (see Material and Methods). In ABAE cells, the percentage of ApoA-I and HDL-mediated cholesterol effluxes was significantly slightly decreased when compared with control condition (Figures 7A,C) whereas no significant differences were reported in SMCs when compared with control condition, except for Rameβ condition (Figures 7B,D) in which an increase in the percentage of ApoA-I-mediated cholesterol efflux was observed. These results suggest that when the intracellular cholesterol content decreased (due to CD treatment in this case), the associated downregulation of ABC transporters provoked a decrease of the cholesterol efflux in order to maintain a quite stable and continuous flow of cholesterol exchanges between cells and media/plasma.

### Effect of T0901317 on RCT

**Figure 8:**
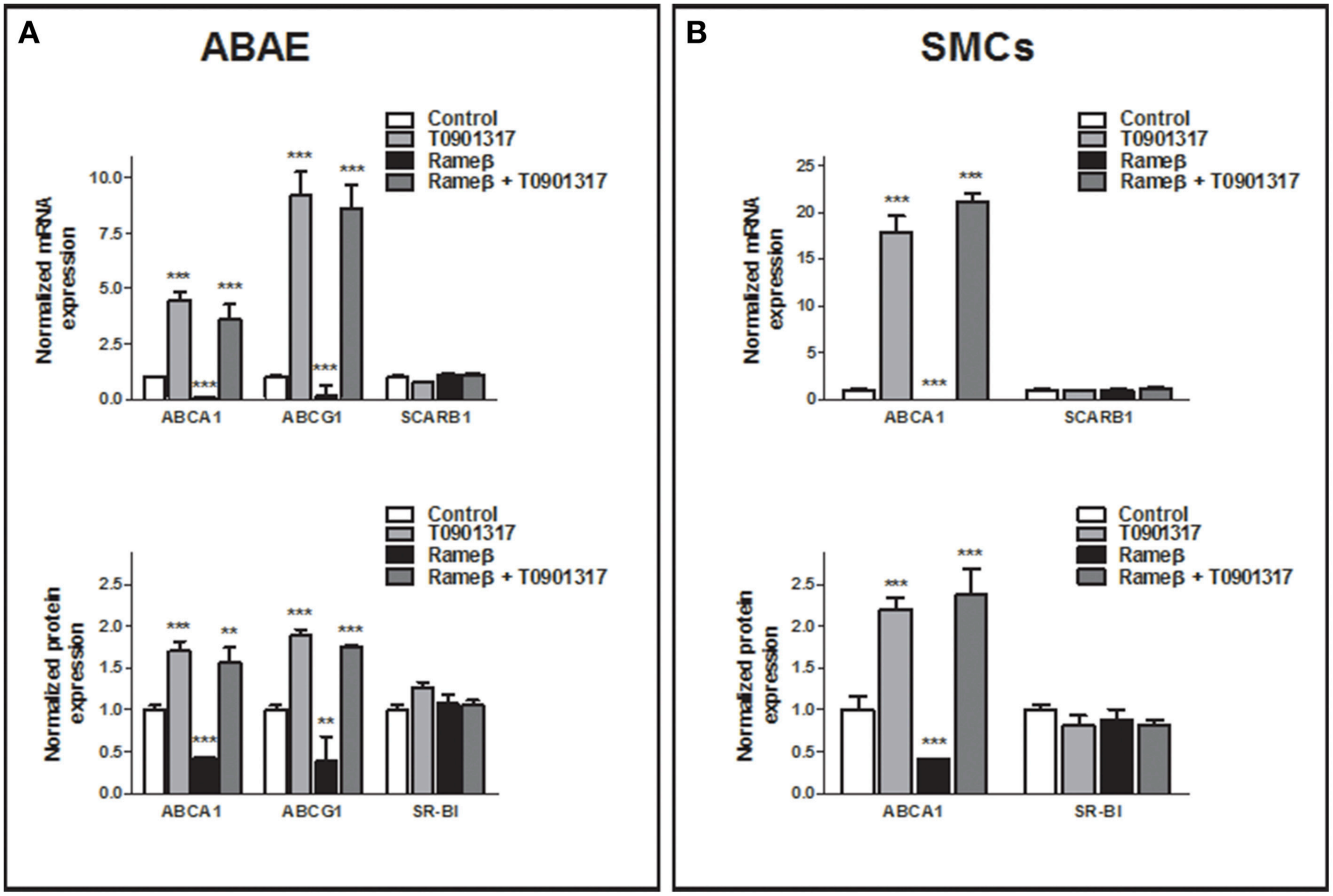
**Effect of 10 μM T0901317 on ABCA1, ABCG1, and SR-BI expression in ABAE (A) and SMCs (B) treated or not with Rameβ**. Cells were first labeled as previously described and then treated with 1 mM of Rameβ supplemented or not with 10 μM T0901317. Transcriptional and protein expression of ABCA1, ABCG1, and SR-BI were studied by real-time PCR and immunoblot in ABAE **(A)** and SMCs **(B)** and compared with control (DMSO treatment, open bar). For mRNA study, each bar represents the mRNA expression normalized against the housekeeping gene β*-ACTIN* ± SEM (*n* = 6). For protein expression study, β-ACTIN was used as the loading control and results correspond to the optical density measured in two independent experiments performed in triplicate. Control condition is used as the reference level and set to 1. A one-way ANOVA test followed by Dunnett's test for multiple comparisons was applied with ^**^p < 0.01; ^***^p < 0.001 compared with control condition.

**Figure 9:**
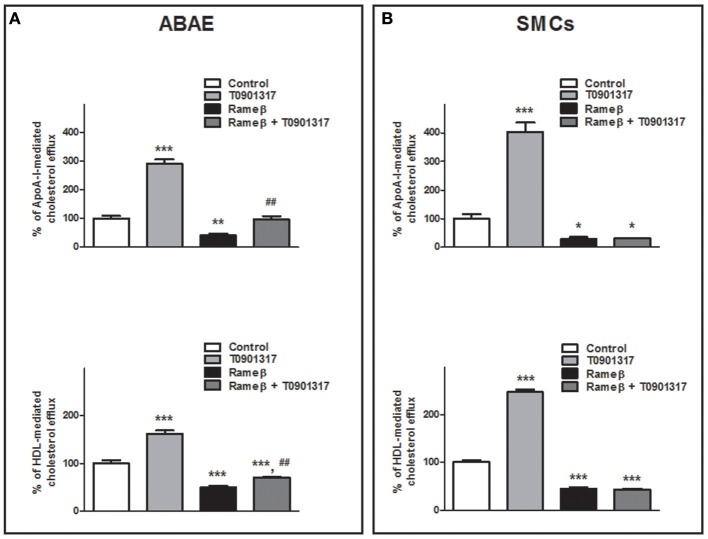
**Effect of 10 μM T0901317 on cholesterol efflux to ApoA-I and HDL in ABAE (A) and SMCs (B) treated or nor with Rameβ**. Cells were first labeled with [^3^H]-cholesterol as previously described and incubated in the presence of 1 mM Rameβ, 10 μM T0901317 or both during 24 h. Then, [^3^H]-cholesterol released in the medium from both cell types was measured 6 h after medium supplementation with either ApoA-I (20 μg/mL) or HDL (50 μg/mL). Data are expressed as the mean ± SEM (in DPM/μg of proteins, *n* = 6 from two sets of experiments). Control condition (without any treatment) is used as the reference level and set to 100%. Statistical analysis: a one-way ANOVA followed by Dunnett's test for multiple comparisons in which ^*^p < 0.05; ^**^p < 0.01; ^***^p < 0.001 compared with the untreated condition (Control condition, open bars) and in which ^*##*^*p* < 0.01 when Rameβ + T0901317 condition was compared with Rameβ condition.

To determine whether the synthetic LXR-agonist T0901317 would partially reverse the effect of CD treatment observed in Figure 6 for ABAE and SMCs, these cells were treated with both 1 mM Rameβ and 10 μM T0901317. In ABAE cells, T0901317 treatment increased mRNA and protein expression levels of ABCA1 and ABCG1 as well as together with Rameβ treatment compared with control condition (DMEM alone) (Figure 8A). As previously reported by us in other cell types, the expression of SR-BI was not modified by the LXR-agonist treatment (Saint-Pol et al., 2012, 2013) (Figure 8A). Then, LXR-treatment induced higher cholesterol effluxes to ApoA-I and HDL in ABAE (289 and 162% when compared with the control condition (100%) respectively, Figure 9A). As reported in Figures 6A,C, ABAE treated with Rameβ showed a decrease in the cholesterol release to ApoA-I and HDL, however, cholesterol efflux get significantly higher when cells were treated together with Rameβ and T0901317 (40% when treated with rameβ alone vs. 96% when treated with Rameβ + T0901317 in the presence of ApoA-I and 50% when treated with rameβ alone vs. 72% when treated with Rameβ + T0901317 in the presence of HDL) (Figure 9A). In SMCs, ABCA1 expression was increased whereas SR-BI expression was not (Figure 8B). As observed in ABAE, treatment of SMCs with T0901317 resulted in an increase in cholesterol release to ApoA-I and HDL (404 and 248%, respectively) (Figure 9B). As previously described, when these cells were treated with Rameβ a significant decrease in the cholesterol efflux to ApoA-I and HDL was observed (30 and 44% when compared with the control condition, respectively) however the addition of T0901317 to Rameβ treatment did not increase significantly cholesterol release in CD-depleted SMCs (31 and 45%, respectively).

## Discussion

Atherosclerosis is characterized by inflammatory processes leading to an abnormal accumulation of cholesterol in the artery vessel wall. This event results from an imbalance between the accumulation and the efflux of cholesterol in macrophages, endothelial cells, and SMCs (Kruth, 2001). In health, these cells do have specific pathways involved in cholesterol export/transfer to extracellular HDL via the so-called RCT. This process remains the major pathway for regulating cellular cholesterol pools and is mediated by proteins expressed by most cell types: two members of the ATP-binding cassette (ABC) transporter family, ABCA1 and ABCG1, and SR-BI (Phillips, 2014). However in atherosclerotic arteries it has been reported that macrophages, SMCs and arterial endothelial cells display altered expressions of these ABC transporters. Although some studies report an up-regulation of ABCA1 and ABCG1 expression, others show a decreased SR-BI and ABC transporter expressions in atherosclerotic arteries when compared with intact or less affected arteries (Albrecht et al., 2004; Forcheron et al., 2005; Soumian et al., 2005; Choi et al., 2009; Ishikawa et al., 2009). Nevertheless, it is now well-approved that ABCA1, ABCG1, and SR-BI represent three major key players in atherosclerosis. Modulating their level of expression and/or functionality, which in turns would affect the amount of plasmatic HDL, is now considered as a valuable and promising therapeutic approach to treat atherosclerosis (Tuteja and Rader, 2014). In line with these considerations, recent treatments with KLEPTOSE® CRYSMEβ has shown to improve atherogenesis in ApoE^−∕−^ mice by ameliorating plasmatic HDL amounts and by abrogating some inflammatory processes (Montecucco et al., 2015). This molecule belongs to the cyclodextrin (CD) family whose members are well-known to interact with cell membranes and to extract their membrane cholesterol. In their study, Montecucco and colleagues have essentially investigated the effects of KLEPTOSE® CRYSMEβ on the immune response and inflammatory processes. They observed that both atherosclerotic plaque size and T lymphocyte content are reduced upon CD treatment, highlighting the fact that KLEPTOSE® CRYSMEβ reduces the Th1-mediated immune response. However, the authors have not investigated the impact of this CD on the SMC and aortic endothelial cell RCT. Therefore we investigated the effects of a number of CDs differentially methylated, including KLEPTOSE® CRYSMEβ, on cholesterol accumulation, RCT and HDL genesis in both SMCs and aortic bovine endothelial cells (ABAE).

Overall, our results suggest that RCT occurs in a different manner in SMCs as compared with endothelial cells. In the same culture conditions, we observed that the cholesterol load was higher in SMCs than in ABAE. Subsequently, this load resulted in a higher cholesterol efflux from SMCs compared to ABAE. However, when correlated with the cholesterol load, the rate of HDL-mediated cholesterol efflux was higher in ABAE than in SMCs (represented 16.20 ± 1.87% and 10.90 ± 1.3% for ABAE and SMCs, respectively) demonstrating a better ability of ABAE cells to export cholesterol to HDL particles. In accordance with these results, it was previously reported that, contrary to SMCs, endothelial cells from arteries exhibit few lipid accumulation however the reasons remain unknown (Phillips, 2014). Therefore such phenomenon could be explained by the fact that aortic endothelial cells express both ABCA1 and ABCG1 whereas SMCs only express ABCA1, no ABCG1, thus decreasing the ability of SMCs to expel the cellular cholesterol. In fact, ABCG1 is known to be expressed in endosomes in which it promotes cholesterol transfer to the plasma membrane (Tarling and Edwards, 2011). Therefore it is likely that the lack of ABCG1 expression contributes to entrap cholesterol in SMCs. Interestingly, this specificity was also observed in other vascular beds such as in the brain vasculature in which brain pericytes and cerebral SMCs do not express ABCG1 whereas brain capillary endothelial cells do (Gosselet et al., 2009; Saint-Pol et al., 2012).

The difference between SMCs and ABAE in their cholesterol metabolism was also observed after cholesterol depletion by CDs as well as after T0901317 treatment. The amount of extracted cholesterol by each tested CD was quite similar in ABAE and in SMCs. However, endothelial cells compensated their cholesterol depletion by decreasing their cholesterol efflux whereas this process was very much less visible in SMCs. To gain insight into the link between cholesterol loss and cholesterol efflux, we evaluated the initial cellular cholesterol load at the start of the efflux experiment and the percentage of cholesterol efflux was calculated taking into account this value. We observed that the percentage of cholesterol efflux was slightly but significantly decreased in ABAE but not in SMCs. Our results also suggest that this reduction in cholesterol content due to CD treatment induced a decrease in cholesterol efflux probably in order to maintain an almost stable and continuous flow of cholesterol exchanges between cells and media according to their intracellular cholesterol content.

Our study also reports that CD-mediated cholesterol extraction decreased the transcriptional expression of protein-encoded genes regulating the RCT, such as ABCA1 and ABCG1 but did not modulate SR-BI expression. To our knowledge the links between membrane cholesterol content and ABC transporter expression remains unclear although it is likely that cholesterol extraction may disrupt lipid rafts, which in turn modify cell signaling pathways and cell physiology (Maclellan et al., 2005; Portilho et al., 2012; Possidonio et al., 2014; Shirao et al., 2015). One of the possible pathways impacted by lipid raft disorganization could be the nuclear LXR pathway that controls ABCA1 and ABCG1 expression in several cell types. When LXRs are stimulated by natural or synthetic ligands, or when cells are loaded with cholesterol, transcriptional expressions of ABCA1, and ABCG1 are induced and lead to an increase in cholesterol release to ApoA-I, ApoE, or HDL (Hirsch-Reinshagen and Wellington, 2007; Saint-Pol et al., 2012, 2013; Gosselet et al., 2014; Koldamova et al., 2014). In our case, the cholesterol depletion induced by CD treatment decreased the expression of both ABC transporters correlating with a decrease in the cholesterol release from cells and more strikingly from ABAE. Thus, it is likely that the action of CDs inhibits LXR signaling pathway. To test this hypothesis and to better characterize the link between ABC transporter expression and cholesterol release/content, we tested the effect of the synthetic LXR agonist T0901317 on ABAE and SMCs. As expected, we observed an increase in the ABCA1 and ABCG1 expressions as well as an increase in the ApoA-I- and HDL-mediated cholesterol efflux from ABAE upon T0901317. When these endothelial cells were concomitantly depleted in cholesterol by CD treatment, these processes also occurred. However, in SMCs the increase in ABCA1 expression was correlated with an increase in their cholesterol efflux only when cells were treated with T0901317 in the absence of CD. This discrepancy confirms our previous observation stating that cholesterol metabolism is different in ABAE and SMCs. These finding needs further investigations to fully understand why LXR activation after a CD-treatment do not induce any effect on cholesterol release in SMCs. One possibility is that such process may represent a safety mechanism in order to limit the cellular cholesterol outflow and its resulting cell death.

Our study also raises some questions regarding the exact role of ABC transporters in RCT mechanism in ABAE and SMCs. In SMCs, although ABCA1 expression was enhanced by T0901317 treatment together with Rameβ treatment, cholesterol efflux was not rescued. Additionally in ABAE, T0901317 treatment induced an increased in both ABCA1 and ABCG1 expressions to the same degree in addition to Rameβ treatment or not, but cholesterol efflux was only partially rescued. Therefore, we can clearly assume that other unidentified transporters involved in cholesterol exchanges would mediate this process in these cell types or that transporter relocalization would occur and influence cholesterol efflux.

Although we report a decrease in HDL synthesis by ABAE and SMCs, a recent study from Montecucco and colleagues reports an increase in plasmatic HDL content in *ApoE*-depleted transgenic mice after intraperitoneal injections of KLEPTOSE® CRYSMEβ (Montecucco et al., 2015). We cannot exclude that this discrepancy might be due to the fact that the cells we used in our *in vitro* studies were not depleted in *ApoE* gene expression or whether these cells were not cholesterol-loaded. Moreover, different regulation, expression or functionality of ABCG1, ABCA1, and SR-BI occurring in the liver of *ApoE*-depleted mice as compared with the wild type situation would also explain the increase of HDL reported *in vivo* (Montecucco et al., 2015).

Importantly, independently of the lipid and protein contents of cell membranes between SMCs and ABAE, each CD investigated in this study displays similar cholesterol depletion abilities in both cell types. These observations suggest that the percentage of extracted cholesterol rather depends on the methylation degree of the CDs than in the cell membrane composition or cell content. This cholesterol extraction process induced by CDs is not well-characterized yet but seems to depend on the CD distribution on the cell surface (López et al., 2011). It has recently been suggested that CDs are able to passively diffuse into the lipid bilayer by pointing their opened secondary rim toward the lipidic polar groups and then remain anchored to the phosphate and glycerol-ester groups via hydrogen bond formation (Khuntawee et al., 2015). Noteworthy, we observed that the methylation degree greatly influences the ability of CDs to extract cholesterol from both cell types. We clearly demonstrated that β-CD and Rameβ represent the most efficient CDs able to extract membrane cholesterol whereas KLEPTOSE® CRYSMEβ and Methyl-β-CD extracted intermediate amounts of cholesterol. This is of prime importance as aberrant cellular cholesterol depletion causing cell deaths are widely reported in the literature upon CD treatment (Kiss et al., 2010; Mahammad and Parmryd, 2015). Further investigations based on the amelioration of design/targeted methylation of the CDs would greatly improve and optimize their effects at the cellular level.

To conclude, our study provides new evidence that methylated β-CDs can efficiently interfere with the pathological process of atherosclerosis by depleting cholesterol content in cells. In response to this cholesterol depletion, cells may modulate ABCA1 and ABCG1 gene expression, regulating in turn their own cholesterol content and vice versa. These results are of great importance in the rational design of efficient CD-based therapeutic approaches, nonetheless, further *in vitro* and *in vivo* studies are still necessary to evaluate the potential of β-CDs to treat atherosclerotic patients.

## Author contributions

CC, DH, LF, and FG designed the study and interpreted the results. Cell isolation, cell culture, and cell death assays were performed and analyzed by CC, DH, and MB, EM, DW, MS, and XP. generated the cyclodextrin family which was deeply characterized by JH, ST, and HB. CC and MB performed mRNA and protein extractions and RT-PCR and immunoblotting experiments. Cellular cholesterol efflux experiments were performed by CC, FG, LF, and CC wrote the manuscript with input from the other authors. Correspondence and requests for materials should be addressed to FG or DW.

### Conflict of interest statement

The authors declare that the research was conducted in the absence of any commercial or financial relationships that could be construed as a potential conflict of interest. XP, MS, and DW are paid employees of Roquette Frères.
